# Pharmacoepigenomic Interventions as Novel Potential Treatments for Alzheimer’s and Parkinson’s Diseases

**DOI:** 10.3390/ijms19103199

**Published:** 2018-10-16

**Authors:** Oscar Teijido, Ramón Cacabelos

**Affiliations:** 1 EuroEspes Biomedical Research Center, Institute of Medical Science and Genomic Medicine, 15165 La Coruña, Spain; rcacabelos@euroespes.com; 2 Chair of Genomic Medicine, Continental University Medical School, Huancayo 12000, Peru

**Keywords:** Alzheimer’s disease (AD), DNA methyltransferase inhibitors/activators, histone acetyltransferase activators/inhibitors, Histone deacetylase inhibitors, histone methyltransferase inhibitors, histone demethylase inhibitors, non-coding RNAs, Parkinson’s disease (PD), sirtuin activators

## Abstract

Cerebrovascular and neurodegenerative disorders affect one billion people around the world and result from a combination of genomic, epigenomic, metabolic, and environmental factors. Diagnosis at late stages of disease progression, limited knowledge of gene biomarkers and molecular mechanisms of the pathology, and conventional compounds based on symptomatic rather than mechanistic features, determine the lack of success of current treatments, including current FDA-approved conventional drugs. The epigenetic approach opens new avenues for the detection of early presymptomatic pathological events that would allow the implementation of novel strategies in order to stop or delay the pathological process. The reversibility and potential restoring of epigenetic aberrations along with their potential use as targets for pharmacological and dietary interventions sited the use of epidrugs as potential novel candidates for successful treatments of multifactorial disorders involving neurodegeneration. This manuscript includes a description of the most relevant epigenetic mechanisms involved in the most prevalent neurodegenerative disorders worldwide, as well as the main potential epigenetic-based compounds under investigation for treatment of those disorders and their limitations.

## 1. Introduction

Brain disorders with a vascular and/or neurodegenerative component affect one billion people worldwide, according to the World Health Organization. Most of these pathologies share an onset of dementia. Disability caused by dementia increases dramatically with aging, by affecting 9 per 1000 of the population aged 65 to 74 years to 83 per 1000 in the population over 85 years old [1]. Alzheimer’s disease (AD) is the major cause of dementia in Western countries, affecting 45 to 60% of the population, followed by vascular dementia and mixed dementia with prevalences of 30 to 40% and 10 to 20%, respectively [2,3]. Alzheimer’s disease (AD) is a complex polygenic disorder defined clinically by a progressive neurodegenerative disorder, resulting in a gradual, irreversible loss of memory and cognitive function and neuropathologically by gross atrophy of the brain and the accumulation of extracellular amyloid plaques and intracellular neurofibrillary tangles. Early stages of AD are characterized by mild cognitive impairment and several histopathological hallmarks including neuritic plaques, neurofibrillary tangles, and loss of basal forebrain cholinergic neurons. AD progression results in senile plates and neurofibrillary tangles corresponding to β-amyloid (Aβ) aggregation and hyperphosphorylation of tau protein, respectively, which results in loss of synapses, neuronal degeneration, and subsequent memory impairment, dementia, and functional decline [2,3,4]. Parkinson’s disease (PD) is the second in the ranking of the most common neurodegenerative disorders, after AD, affecting 2% of the population over 60 years of age in the world [5], and involves genetic, environmental, cerebrovasular, and epigenetic factors [6,7,8,9,10]. PD is a complex neurodegenerative disease characterized by progressive degeneration of dopaminergic neurons in the substantia nigra pars compacta and the formation of intracytoplasmatic inclusions made of accumulations of α-synuclein known as Lewy bodies [11,12]. Clinical features of PD include rigidity, resting tremor, bradykinesia, and postural imbalance.

Neurodegeneration process begins with a series of earlier events, affecting cell development, metabolism, and axonal transport, which progressively lead to a massive cell death rate. Brain often compensates those premature features that remain rather asymptomatic. The lack of success of the current treatment strategies relies on our still limited knowledge of the pathogenic genomic variants. Importantly, currently detected polymorphic variants in pathogenic genes associate directly with a very low rate of Alzheimer’s or Parkinson’s disease patients. Personalization of treatments according to individual pharmacogenomic profiles would also increase the rate of success and reduce unnecessary costs. In addition, detection of the first symptomatic features normally occurs after a high rate of cell death and damaged tissue, which significantly affect brain function and hinders potential treatments. In this regard, novel epigenetically-based treatments are gaining a great interest as a potential novel treatments for complex multigenic neurodegenerative diseases [3,13,14,15,16,17].

Complex diseases often result from the interplay of genetic variants (genomics) and the environmental influence on gene function (epigenomics). The epigenetic machinery controls metabolic pathways by regulating gene expression through chemical and structural modifications on the genome, such as DNA/RNA methylation, chromatin structure, and non-coding RNA binding. The study of the epigenetic gene regulation might allow the identification of early biomarkers corresponding to previously undetectable features in the progression of neurodegeneration. In addition, these epigenetic aberrations may be restored with the use of appropriate epigenetic-based therapies. In this regard, during the last decade, scientists have been highly motivated in the search of epigenetic aberrations that occur during presymptomatic disease stages in order to establish novel treatment approaches using compounds (epidrugs) which target the epigenetic aberrations occurring during the progression of neurodegenerative processes [2,13,18,19,20,21]. These novel approaches will potentially reduce or delay the onset of these diseases, which would improve or elongate life quality of the patients, and would reduce disease management costs. Despite of the promising results on cell and animal models, epidrugs must fulfill certain requirements for the proper evaluation of their efficacy and safety in clinical trials, including (i) more physiological IC_50_ ranges and efficient drug delivery strategies; (ii) more specific targets; and (iii) personalized treatments according to the individual pharmacogenomic and pharmacoepigenomic profiles.

## 2. Current Gene Targets and Pharmacological Treatments for Alzheimer’s and Parkinson’s Diseases

### 2.1. Alzheimer’s Disease (AD)

The complexity of AD pathogenesis relies on the combination of genetic, epigenetic, and environmental factors. Current research accumulates data from over 600 single-nucleotide polymorphisms (SNPs), as well as Mendelian and mitochondrial mutations, in genes potentially associated with AD progression [13,22,23,24]. Mendelian mutations affect AD pathogenic genes, including presenilins (*PSEN1* and *PSEN2*), Aβ-precursor protein (*APP*), apolipoprotein E (*APOE*), and the alpha-2-macroglobulin (*A2M*).

APP cleavage, Aβ clearance, and microtubule stability regulated by phosphorylation of microtubule-associated tau protein, are key targets of AD progression. Presenilins are important determinants for the β-secretase-mediated APP cleavage. Polymorphic variants on *PSEN1* and *PSEN2* genes, detected in some AD patients, correlate with an impaired APP cleavage and Aβ aggregation into senile plates. Polymorphisms in the gene encoding the microtubule-associated protein tau (MAPT) promote tau protein hyperphosphorylation which results in microtubule destabilization leading to neurofibrillary degeneration [2,3,23,25]. Polymorphic variants in the gene encoding apolipoprotein E (*APOE*), which associate with hypercholesterolemia and vascular disorders, constitute one of the most relevant genetic hallmark s of AD. Presence of the *APOE-ε4/ε4* haplotype represents a 60 to 80% probability of an early AD onset [25,26,27]. Although the molecular mechanisms are not clear, several studies associate *APOE-ε4* with an impaired APP metabolism leading to Aβ aggregation promoting tau hyperphosphorylation resulting in the formation of fibrillary tangles, as well as lipid metabolism and transport impairment and oxidative and neuroinflammatory processes leading to a massive cell death rate [25,26,28,29,30]. Importantly, the presence of *APOE-ε2*, tightly linked to pathologies with a vascular component, is nevertheless protective against dementia [25,26]. The *A2M* gene, encoding for the alpha-2-macroglobulin (a protease inhibitor), is also localized in amyloid plaques and interacts with Aβ and APOE. The polymorphism 2998 G > A (rs669) in homozygosis increases the risk for the onset of AD by 4-fold compared with the general population [23,25,26].

Most current pharmacological approaches for AD treatment rely on promoting cholinergic synapses, reducing neuronal cytotoxicity, or preventing the formation of senile plates [2,13,31,32,33,34,35]. Despite numerous attempts during the last thirteen years, the only five drugs approved by FDA tacrine, donepezil, rivastigmine, galantamine, and memantine, demonstrated limited success. AD-related impaired memory and learning tasks as well as lack of attention, associate with a loss of cholinergic neurons [31,32]. Therefore, the first pharmacological strategies relied on the generation of cholinesterase inhibitors in order to promote acetylcholine levels at cholinergic synapses. Unfortunately, the positive effects of these compounds were rather controversial [2,13,33]. The high affinity antagonist of glutamatergic *N*-methyl-d-aspartate (NMDA) receptors, memantine, is a current alternative strategy for patients with moderate or severe stages of the disease. Memantine reduces neuronal excitotoxicity by inhibiting the prolonged influx of Ca^2+^ ions from extrasynaptic receptors. Nevertheless, efficacy of this drug is under debate [2,13,34,35]. Lack of success of alternative approaches based on preventing the formation of senile plates by β-secretase inhibitors or immunotherapy relied on undesirable side effects on detriment of the scarce beneficial effects [13].

### 2.2. Parkinson’s Disease (PD)

Recent studies provide explanations about the implications of α-synuclein in PD at the molecular level. It has been recently established the interaction of α-synuclein with mitochondrial membranes [36,37,38,39,40] and its implication in mitochondrial impairment leading to cell death [41,42]. α-synuclein affects Complex I [38] and Complex IV [43] of the mitochondrial respiratory chain, leading to a bioenergetic dysregulation, resulting in ROS production and cell death. Experiments in vitro and in yeast mitochondria corroborate these results finding that α-synuclein was able to translocate from cytosol to the mitochondrial inner membrane through the voltage-dependent anion channel (VDAC) and target the mitochondrial respiratory chain [44].

Besides the *SCNA* gene, which encodes α-synuclein, over 100 other pathogeneic genes may be involved in PD, from which 15 PD loci (*PARK1-15*) as well as other loci might be a direct cause of the disease [45]. Mutations in synuclein-alpha (*SNCA*), parkin 2 (*PARK2*), PTEN-induced putative kinase 1 (*PINK1*), parkin 7 (*PARK7*, *DJ1*), and leucine-rich repeat kinase 2 (*LRRK2*) genes are associated with the genetic etiology of PD, whereas other loci, such as, microtubule-associated protein tau (*MAPT*), *spatacsin*, polymerase (DNA) gamma, catalytic subunit (*POLG1*), glucosylceramidase beta (*GBA*), and ataxin (*SCA1*, *SCA2*), might be susceptibility genes associated with sporadic PD, normally associated with toxic or environmental exposure [8,46].

The loss of dopaminergic neurons during development of PD results in concomitant loss of dopamine in the affected areas (especially the nigrostriatal system) which is manifested with classic motor symptoms (resting tremor, rigidity, bradykinesia, postural instability, and slowness of movements which ends up in muscle atrophy), and other non-motor symptoms (depression, obsessive compulsive behavior, sleep disturbance, and cognitive impairment, among others). Current pharmacological treatments for PD are based on restoring the dopamine levels using different strategies: (i) increase dopamine availability by treatments with dopamine precursors, such as L-DOPA (levodopa), or dopaminergic agonists (amantadine, apomorphine, bromocriptine, lisuride, cabergoline, pergolide, pramipexole, ropinirole, and rotigotine) and (ii) inhibition of dopamine catabolism or degradation, by using monoamine-oxidase B (MOB) inhibitors, such as rasagiline and selegiline, or catechol-*O*-methylatransferase (COMPT) inhibitors, such as entacapone and tolcapone. Unfortunately, all these pharmacological treatments only provide a symptomatic relief rather than stopping or delaying the progression of the disease. Furthermore, the chronic administration of antiparkinsonian drugs currently induces the “wearing-off phenomenon”, with additional psychomotor and autonomic complications [7,17].

Combination of current drugs with novel compounds, especially bioproducts seem to reduce these clinical complications and provide dopaminergic neuroprotection in order to enhance dopaminergic neurotransmission and reduce premature neurodegeneration [17].

## 3. Main Epigenetic Hallmarks of Neurodegeneration

The analysis of the whole genome sequencing unveils a wide range of gene variants which facilitate the diagnosis of a number of complex, multigenic disorders. These polymorphic variants and gene mutations also serve as targets for developing more accurate and less expensive treatments. Nevertheless, genetic factors define only partially the onset of neurodegenerative diseases, which normally arouse due a complex interplay of genetic and epigenetic mechanisms. The strict epigenetic regulatory processes result in the control of gene expression in response to the metabolic demands of the organism. Epigenetic mechanisms regulate gene expression at both, transcriptionally and post-transcriptionally levels. While DNA methylation status and modulation of chromatin structure, mediated by ATP-dependent chromatin regulator complexes (ATP-CRCs) and post-translational histone modifications, exert a transcriptional control, non-coding RNAs suppress gene expression post-transcriptionally [47].

Neurophysiologic mechanisms integrated in upper level complex processes, such as memory acquisition, learning, or motor coordination are, to a large extent, epigenetically regulated [48,49,50]. Therefore, alterations in this meticulously controlled epigenetic machinery increase the risk for onset of disorders that involve mental decline, memory and motor impairments, brain deterioration, and neurodegeneration. These epigenetic aberrations target genes directly related to the pathogenesis, such as those modulating synaptic plasticity, immune response, cell development, and apoptosis, and also genes indirectly involved in the disease [2,18,19,21].

### 3.1. DNA Methylation

DNA methylation consists in the incorporation of methyl groups into cytosine molecule, normally located at CpG islands where CG content is greater than 60%. The level and location of DNA methylation affect gene function in different manners. Levels of methylation at the promoter regions affect gene expression. Generally, the higher is the level of methylation at the promoter region, the lower is the expression of this gene, and vice versa. Promoter hypermethylation promotes the binding of transcription repressors or inhibits transcription factors leading to a reduced gene expression [18,51,52]. Three main DNA methyltransferases (DNMTs) are responsible for DNA methylation process in mammals. DNMT3a and DNMT3b add methyl groups to new unmethylated cytosines, whereas DNMT1 maintains the methylated status [53,54]. As a counterpart, DNA demethylases reduce DNA methylation levels and promote transcription. Three enzyme families mediate DNA demethylation process: (i) the ten-eleven translocation (TET) family, which converts of 5-methyl-cytosine (5mC) into 5-hydroxymethyl-cytosine (5hmC); (ii) the AID/APOBEC family, acting as mediators of 5mC or 5hmC deamination; and (iii) the BER (base excision repair) glycosylase family involved in DNA repair [19].

#### 3.1.1. Global DNA Methylation and Neurodegeneration

The two most prevalent neurodegenerative disorders worldwide, Alzheimer’s (AD) and Parkinson’s diseases (PD), share a reduced DNA methylation in brains and blood from animal models and human subjects [2,18,46,55,56,57,58,59,60,61,62,63,64]. Reduced expression of DNMTs and impaired Vitamin B activity are the main players of this global hypomethylation. Vitamin B complex (vitamins B2, B6, B12, and folate) displays important brain protective benefits and restores the proper DNA methylation levels by promoting homocysteine (Hcy) methylation by the *S*-adenosyl-l-methionine-dependent methyltransferase (SAMe) [18,65]. Indeed, both AD [66,67,68,69] and PD [62,63,64] associate with reduced levels of SAM, which result in a defective methylation of Hcy, which promotes promoter demethylation. Vitamin B deficiency also induces hypomethylation leading to overexpression of specific genes involved in AD pathogenesis. In this regard, deprivation of folate and vitamins B6 and B12 led to hypomethylation and overexpression of the β-secretase 1 (*BACE1*) and presenilin 1 (*PSEN1*) genes in cell cultures, transgenic AD animal models, and post-mortem brains of AD patients [70,71,72,73,74]. This excessive β-secretase activity resulted in impaired APP cleavage and promoted Aβ aggregation into senile plates. Some studies also suggest a link between the formation of alpha synuclein protein (α-Syn) aggregates, a PD hallmark, with reduced SAMe levels [75,76]. According to these studies, defective SAMe activity may reduce methylation levels at the promoter region of the α-Syn-encoding gene (*SCNA*), which would promote the expression and aggregation of α-Syn.

Contrary to AD and PD, the epigenetic pattern of the Amyotrophic Lateral Sclerosis (ALS) involves a general DNA hypermethylation [77] enforced by increased DNMT expression [78,79] and impaired demethylation machinery [80]. According to Al-Chalabi and colleges [81], approximately 10% of ALS forms are familial and caused by gene mutations whereas 90% are sporadic, i.e., influenced by surrounding environment [81].

#### 3.1.2. Gene Specific Methylation and Neurodegeneration

Most aberrant DNA methylation patterns leading to neurodegeneration target pathogenic genes directly involved in the disease, genes involved in neuroinflammatory pathways, and genes related to neurodevelopment and synaptic processes.

Despite of the importance of β-secretase activity and APP cleavage in the progression of AD [2,3,23,25], except for some exceptions [82,83,84,85], most of the studies find no direct correlation between *APP* methylation and AD progression [46,55,56,57,58,59,86]. Hyperphosphorylation of protein tau is one of the molecular hallmarks of AD-related neurodegeneration. Excessive protein tau phosphorylation reduces the binding affinity of this protein to cytoskeleton which detaches and accumulates into free aggregates forming neurofibrillary tangles (NFTs). Protein tau detachment also leads to a concomitant destabilization of cytoskeleton and cell structure. Vitamin B deficit in AD patients reduces methylation of the glycogen synthase kinase 3β gene (*GSK3β*) at the promoter region, which promotes the expression of this protein kinase that induces tau phosphorylation, NFT aggregation, loss of cytoskeleton integrity, and cell death [87]. Promoter hypomethylation also induces the expression of genes involved in cell death and neuroinflammation, such as bridging factor 1 (*BIN1*), complement receptor 1 (*CR1*), the CD33 molecule (*CD33*), and the tumor necrosis factor (*TNF-α*) [2,21,56,88]. However, promoter hypermethylation reduces the expression of the sortilin-related receptor (*SORL1*) and neprylisin (*NEP*) genes involved in the Aβ degradation and clearance. Sanchez-Mut et al. [89] found that hypermethylation in promoter regions of the thromboxane A2 receptor (*TBXA2R*), sorbin and SH3 domain containing 3 (*SORBS3*), and spectrin beta 4 (*SPTBN4*) genes in AD animal models and human subjects. Authors suggest that the activation of the cyclic AMP response element-binding protein (CREB) pathway and the axon initial segment might contribute to the pathogenesis of AD [89]. 

Although *APOE* gene haplotypes are among the most reliable biomarkers for AD diagnosis, information available about the epigenetic modulation of this gene is scarce. Some studies suggest that the C > T transition in the 3’-CpG island, which is specific of *APOE-ε4*, might prevent methylation of this site and promote *APOE-ε4* expression in AD patients [18,71,90].

Genome wide association studies found a direct implication of methylation status of α-synuclein and development of PD. The putative gene promoter, located in the intron 1 of *SCNA* gene, was significantly hypomethylated in blood and brain samples from PD patients as compared to controls [91]. This hypomethylation was associated with the overexpression of α-synuclein and protein aggregation leading to PD [7]. This hypomethylation/overexpression is observed in substantia nigra, putamen, and cortex of sporadic PD cases [62,92].

Other genes were also found epigenetically regulated in PD. Increased *TNF-α* levels are associated with neuroinflammation and dopaminergic cell death in PD. Therefore, the higher vulnerability to *TNF-α* regulation found in dopaminergic neurons suggests the gene promoter is hypomethylated [93]. Importantly, *TNF-α* overexpression is usually detected in the cerebrospinal fluid of PD patients, as *TNF-α* induces apoptosis in neuronal cells [93]. It was recently reported the aberrant expression of clock genes in animal models of PD [94,95]. Methylation level of seven clock gene promoters was analyzed finding a reduced methylation in PD compared to controls [96]. In addition, DNA methylation, among other epigenetic mechanisms, plays an important role in mesodiencephalic dopaminergic neurons, which are severely affected in PD patients [97].

Other studies revealed that methylation aberrations may associate with imprinting mechanisms, such as those responsible for huntingtin overexpression in Huntington’s disease patients [98], or the risk of triggering intergenerational extension or instability of CAG repeat expansions by changes in DNA methylation during epigenetic reprogramming [99,100].

### 3.2. Histone Post-Translational Modifications Affecting Chromatin Remodeling

Chromatin stability and conformation regulates gene expression and silencing of transposable elements, as well as maintains genome integrity. ATP-dependent chromatin regulator complexes (ATP-CRCs) and post-translational histone modifications control chromatin structure.

Histone post-translational modifications alter the chromatin package into a tight (hetrochromatin) or loose (euchromatin) conformation, which affects gene accessibility to the transcription machinery. Histone acetylation, mediated by histone lysine-acetyltransferases (HATs or KATs) reduces the electrostatic interaction between DNA and histones which results in a looser chromatin conformation that allows gene accessibility and thus activates transcription [101,102]. Gcn5-related *N*-acetyltransferases (GNATs), which include GCN5, p300/cAmp-response element binding protein (CBP)-associated factor (PCAF), KAT6-8, CREB-binding protein/CBP (CREBBP/CBP), and EP300 promote histone acetylation [55,101,102,103,104,105,106]. On the other hand, histone deacetylases (HDAC), reduce the level of acetyl groups and thus the negative charge of histones which enhances the electrostatic binding to DNA and promotes a compact chromatin conformation with the subsequent repressed gene transcription [2,18,48,101,102,103,104,105,107]. This conformation limits the access for transcription factors but also for DNA repair machinery, which negatively affects to the number of synapses leading to impaired memory and learning abilities. Disruption of HAT/HDAC equilibrium associates with histone acetylation decay, which increases with aging and drastically declines during AD progression, especially in the temporal lobe of AD patients [108,109] and in AD animal models [48,110]. Overexpression of nuclear EP300 interacting inhibitor of differentiation 1 (EID1) in cortical neurons of AD patients and animal models promotes histone hypoacetylation mediated by inhibition of EP300 and CREBBP [111,112]. HDAC2-mediated acetylation decay located in prefrontal cortex and hippocampus reduced neuroplasticity, as well as downregulated genes involved in learning, memory, and synaptic plasticity in AD mice [48,113]. Some studies also suggest that one of the neurotoxic effects of α-synuclein in PD involves its direct binding to histones, preventing H3 acetylation [114,115]. Indeed, treatment with HDAC inhibitors reduced α-synuclein neurotoxicity in neuroblastoma cells and transgenic Drosophila [114,115,116]. Alternatively, the class III NAD^+^-dependent HDACs, sirtuins (SIRT), promote lifespan and healthy aging by delaying the onset of neurodegenerative processes [21,46,117,118]. Importantly, several studies using animal models indicate that sirtuin expression drifts with aging [119,120,121] as well as with age-related neurodegenerative disorders [122,123].

Histone methylation and demethylation, mediated by histone methylases (HMTs) and demethylases (HDMTs), respectively, also contribute to neurodegenerative progression. Those enzymes have a high specificity as they usually modify one single lysine per histone which may be translated into activation or repression of transcription [19,55,101,102,124,125]. Histone methylations H3K4, H3K36, and H3K79 are associated with the activation of gene expression, whereas methylations at H3K9, H3K27, and H4K20, correspond to gene silencing. Histone methylation has also been associated with DNA repair [1,55,101,126].

Histone methylation levels (H3K14, HeK9me2, among others) increased significantly in young preplaque AD transgenic mice as compared with wild-type mice [110,127,128]. Some studies suggest the role of histone methylation promotes polarization of microglial activation pathways involving dopaminergic cell loss during PD progression. Frequency of classical (M1 phenotype) and alternative (M2 phenotype) activation pathways determines the detrimental or beneficial effects for CNS. Histone demethylase H3K27me3 Jumonji domain containing 3 (Jmjd3) is essential for M2-type activation. Suppression of Jmjd3 magnifies M1-mediated microglial overactivation leading to extensive cell death in substantia nigra in MPTP-intoxicated PD transgenic mice [129]. MPTP-mediated toxicity also reduces H3K4me3 levels in the striatum of mice and non-human primates, which can be reverted through chronic treatment with L-DOPA [130].

### 3.3. Non-Coding RNAs

Differential expression of non-coding RNAs (ncRNAs) modulates gene expression post-transcriptionally. Aberrant expression of a number of ncRNAs alters expression of genes involved in metabolic pathways leading to neurodegeneration.

These regulatory RNAs include long ncRNAs (lncRNAs), which target pathogenic genes directly involved in the disease or epigenetically regulated genes, small interference RNAs (siRNAs), piwi RNAs (piRNAs), and microRNAs (miRNAs). These last ones induce mRNA degradation by binding to the 3′ untranslated region and are the most popular biomarkers for disease diagnosis and progression. Altered expression of miRNAs affects modulation of direct pathogenic genes or those involved in other neurophysiological roles indirectly associated with the disease. A number of cell free blood and cerebrospinal fluid-circulating miRNAs are informative biomarkers of the stage and progression of the disease in vivo, with a rapid and noninvasive liquid biopsy. Circulating miRNAs are thus good candidates as presymptomatic biomarkers and for early diagnosis of the disease.

### 3.4. Epigenetic Regulation of Telomeres

Telomeres are protective structures located at the end of the chromosomes, which contain a number of TTAGGG repeats. Shortening of telomere length increases with aging and age-related and neurodegenerative disorders enhance this process. Several mechanisms mediate the protection of these telomeric regions in order to delay their degradation. The efficiency of these protective mechanisms depends on the length of telomeres, i.e., the number of TTAGGG repeats. Shelterin is a nucleoprotein complex that binds to those repeats and protects DNA from activation of DNA damage pathways. Ability of sheltering binding increases with telomeric length, which is modulated by telomerase activity. Telomerase replaces the missed repeats after each cell division, using an associated RNA molecule, TERC, as a template. However, aging-related telomerase activity decay leads to telomere shortening, resulting in a loss of shelterin’s ability to bind these shorter telomeres and activation of the DNA damage cascade and cell death [131,132].

The epigenetic machinery modulates DNA protection and chromatin structure and stability, which affects telomerase activity and the rate of telomere degradation. Histone hypoacetylation at telomeric regions induces a heterochromatin state which protects DNA at telomeric and subtelomeric regions from activation of DNA damaging pathways [133]. On the other hand, epigenetic alterations, including decreased histone trimethylation of H3K9 and H4K20 or reduced histone dimethylation of H3K79, as well as aberrant DNA methylation, and histone H3 and H4 acetylation at telomeric and subtelomeric regions, would disrupt chromatin stability in those regions and enhance telomere shortening [134,135,136].

Epigenetic mechanisms involving ncRNA also regulate telomere length. A number of subtelomeric loci express lnRNAs named telomeric repeat-containing RNAs (TERRAs) which control both chromatin remodeling at those regions and telomerase-mediated telomere elongation [137,138,139]. TERRA may be aberrantly upregulated by DNA methylation, histone acetylation, or reduced histone methylation at telomeric or subtelomeric regions, which may lead to interference with telomere replication.

## 4. Current Epigenetic-Based Strategies Targeting Neurodegeneration

Available treatments for complex disorders are mostly symptomatic and provide limited beneficial effects on the progression of the disease, and often with a payback of unacceptable side effects. Epigenetic mechanisms unveil many hidden pathological alterations of memory and learning impairment, synaptic loss, and cell death, involved in neurodegenerative processes. Many epigenetic alterations appear in early asymptomatic stages of the disease and are reversible. Thus, the new age attempts of treating these disorders involve the use of epigenetic-based drugs (epidrugs) targeting DNA methylation, chromatin remodeling, and non-coding RNAs as potential candidates for the treatment of these complex polygenic disorders. These drugs include activators and inhibitors of DNA methyltransferases, histone deacetylase inhibitors, sirtuin activators, modulators of histone acetylation and histone methylation, as well as RNA interference analogs.

### 4.1. DNA Methylation Modulators

#### 4.1.1. DNA Methylation Activators

Strategies attempting to restore DNA methylation may re-establish the proper metabolic pathways disrupted by the global DNA hypomethylation associated with the progression of most prevalent neurodegenerative disorders, including AD and PD. In most cases, detrimental Vitamin B- and SAMe-mediated global hypomethylation associates with high levels of homocysteine (Hcy) and S-adenosylhomocysteine (SAH) [18,62,66,67,68,69,75,140], which promotes expression of pathogenic genes. Beneficial effects of some dietary interventions may address this issue, with special focus in diets with high contents of vitamin B complex (B2, B6, B12, and folic acid). Vitamin B and SAMe-mediated DNA methylation involves several signaling pathways affecting folate/methionine/homocystein metabolism, using folate, choline, betaine, methionine, and enzyme cofactors [18,65]. Vitamin B_6_-dependent serine-hydroxymethyltransferase catalyzes the conversion of tetrahydrofolate (THF) into 5,10-methylenetetrahydrofolate (MTHF), followed by the synthesis of 5-MTHF catalyzed by vitamin B_2_-dependent MTHF reductase (MTHFR). 5-MTHF provides the methyl groups for Hcy methylation by cobalamin-dependent methionine synthase, yielding methionine, which is converted to SAM by methionine adenosyltransferase. SAM is responsible for methylation of main macromolecules, including DNA, proteins, phospholipids neurotransmitters, and hormones. Donation of methyl group promotes the synthesis of SAH, which hydrolyzes to Hcy and adenosine by SAH hydrolase. According to the consensus statement, based on the Bradford Hill criteria, elevated levels of total Hcy is a recognizable risk factor for development of dementia and AD in older individuals [141]. Therefore, methylation restorage and brain protective properties of vitamin B, folic acid, and SAMe sites them as good diet supplements for treatment of these disorders [76,142,143,144] (Table 1). Indeed, Vitamin B6 and folate are currently submitted to clinical trials from which three of them are completed in phase II (ClinicalTrials.gov Identifier: NCT01320527), phase III (ClinicalTrials.gov Identifier: NCT00056225), and phase IV (ClinicalTrials.gov Identifier: NCT2457507) to determine whether reduction of homocysteine levels with these dietary interventions would reduce cognitive impairment in AD patients [145,146,147,148] (Table 1). In Sweden and UK, folate and Vitamin B6 are clinically prescribed in patients with elevated levels of total Hcy corresponding to high risk of dementia onset [149,150,151].

#### 4.1.2. DNA Methylation Inhibitors

Hypermethylation of pathogenic genes also promotes neurodegeneration. Therefore, approaches using DNA methylation inhibitors may also be appropriate [2,19,26,53,54,152,153,154,155]. Indeed, several DNMT inhibitors are currently submitted to clinical trials for AD treatment [145,156,157,158,159,160]. DNMT inhibitors are often small molecules and natural products, although nucleoside analogs and ncRNAs also target DNMTs.

The epigallocatechin-3-gallate (EGCG) is the main polyphenol of the green tea (*Camilla sinensis*). EGCG prevents misfolded proteins from fibrillization [156] and restores respiratory rates and membrane potential in isolated mitochondria from hippocampus, cortex, and striatum [157]. In addition, ECGC activates the signaling pathway involving the α7 nicotinic acetylcholine receptor (α7 nAChR) and restores *Bcl2* expression, preventing cell death in Aβ-treated neurons [158]. This DNMT inhibitor is currently under clinical trials in phases II and III to test the effects of this compound on the prevention of Aβ aggregation to toxic oligomers in AD through the direct binding to the unfolded peptide (ClinicalTrials.gov Identifier: NCT00951834) [145] (Table 1). Other natural products include non-nucleosides, such as curcumin derivatives RG-108 and SGI-1027 [54], psammaplins (inhibit both DNMT1 and HDACs [53]), catechins (catechin and epicatechin), and bioflavonoids (quercetin, genistein, and fisetin). Quercetin is one of the components of the Etanercept (Enbrel^®^), which is an approved drug for the treatment of several forms of arthritis when administered by injection. Some studies suggest that perispinally injected Etanercept may modulate certain aspects of the immune system and provide some beneficial effect for people with Alzheimer’s disease. Studies suggest that supplementation with specific nutrients may also have a positive effect in support of cognitive function [159,160]. Etanercept is currently under phase I clinical trials (ClinicalTrials.gov Identifier: NCT01716637) for treatment of mild to moderate AD [37] (Table 1).

Other DNMT inhibitors, such as the nucleoside analogs 5-aza-2′-deoxycytidine (Decitabine) and 5-azacytidine (Azacitidine) and the small molecules hydralazine and procainamide are also potential treatments for neurodegeneration, although they are not yet submitted to clinical trials. However, these epidrugs are currently FDA approved for other prevalent disorders including diverse types of cancer, myelodisplastic syndrome, thalasemia, hypertension, and cardiac arrhythmia [53].

### 4.2. Histone Deacetylase (HDAC) Modulators

#### 4.2.1. Class I, II, and IV HDAC Inhibitors

HDAC inhibitors (HDACi) potentially restore global histone deacetylation, which is a common feature of most neurodegenerative processes. Most of HDACi under development provide beneficial effects at cognitive and memory levels in animal models of AD [2,46,53,55,71,156,161] and PD [162,163,164,165,166,167,168,169]. However, only valproic acid (VPA), nicotinamide, and sodium phenylbutyrate (4-PBA) are currently under clinical trials as epidrugs for treatment of neurodegeneration [145] (Table 1).

The most effective HDACi tested in those models are (i) the short-chain fatty acids, class I HDACi (valproic acid (VPA)) and class I and II HDACis (sodium butyrate (NaB) and sodium phenylbutyrate (NaPBA, 4-PBA)); (ii) the hydroxamic acids, class I and II HDACis (suberoylanilide hydroxamic acid (SAHA, vorinostat) and trichostatin (TSA)); (iii) some benzamides, class I and II HDACi (entinostat (MS-275), W2); (iv) miscellaneous compounds, class I and II HDACi (FRM-0334) and HDAC6 specific inhibitors (Tubacin, Tubastatin A, quinazolin-4-one, (E)-3-(2-Ethyl-7-fluoro-4-oxo-3-phenethyl-3,4-dihydroquinazolin-6-yl)-*N*-hydorxyacrylamide (4b), and *N*-hydroxy-3-(2-methyl-4-oxo-3-phenethyl-3,4-dihydro-quinazolin-7-yl)-acrylamide (3f)); and (v) the class III HDACi or SIRT inhibitor nicotinamide/niacinamide and SIRT activators as resveratrol and derivatives. Other benzamides, cyclic peptides, and ketones showed powerful HDAC inhibition properties and some of them are currently FDA approved for cancer treatment.

Low toxicity of NaB makes this drug tolerable for treatment in animals and humans [169,170,171]. NaB increases the peripheral levels of hypothalamic–pituitary–adrenal axis hormones and glucose. NaB administration reinstated memory and learning activities in transgenic AD mice. In addition, prolonged exposure to NaB improved associative learning and memory in APP/PS1-21-AD transgenic mice, even at a very advanced stage of pathology. This effect might be due to the NaB-mediated histone acetylation in the hippocampus, modifying chromatin structure and enhancing the transcription of genes involved in these tasks [161]. This compound may also be effective in reducing α-synuclein aggregation and toxicity and rescuing cognitive deficits associated with PD in animal models [172,173].

4-PBA constitutes one of the most promising HDACi-based therapeutic agents due to the successful results obtained in animal models. Histone acetylation mediated by 4-PBA, promotes transcription of genes associated with synaptic plasticity and promotes the active form of GSK-3β, preventing tau phosphorylation and restoring memory and learning activities in AD transgenic mice [174]. Among these effects, other studies show an additional Aβ clearance in alternative AD animal models [53]. Combination of 4-PBA with Tauroursodeoxycholic Acid is currently in phase II clinical trials (ClinicalTrials.gov Identifier: NCT03533257) [145] in order to evaluate diverse AD-relevant markers and produce an informative dataset that will allow for evaluation and correlation of imaging-based markers, neurobiological changes, functional measures, and cognitive outcomes. Treatment with 4-PBA also protects dopaminergic neurons, possibly through increased DJ-1 expression and activation of tyrosine hydroxylase promoter in the substantia nigra of mice exposed to the PD-promoting toxic agent 1-methyl-4-phenyl-pyridinium (MPTP) [172,175]. A current phase I clinical trial (ClinicalTrials.gov Identifier: NCT02046434) [145] is attempting to investigate the potential effects of phenylbutyrate on the removal of alpha-synuclein from the brain into the bloodstream. 

The anticonvulsant VPA is a fatty acid originally used as treatment for epilepsy and as a mood stabilizing agent. This is one of the most studied compounds for as a potential treatment for neurodegeneration. VPA treatment promotes the expression of brain-derived neurotrophic factor (*BDNF*) and glial-derived neurotrophic factor (*GDNF*), which play critical roles in the growth, survival, and synaptic plasticity of neurons. In addition, VPA induces the expression of the heat-shock protein Hsp70, accompanied by increased levels of H3 lysine di- and trimethylation (H3K4Me2 and H3K4Me3), which promote the recruitment of HAT p300 [166]. Several studies show the ability of VPA to reduce Aβ production and aggregation in AD cells and animal models, normally by inhibiting GSK-3β-mediated γ-secretase cleavage of *APP* [176,177]. VPA, in combination with NaB and SAHA, promotes histone H4 acetylation, which results in a mitigated memory impairment [178]. VPA is currently submitted to three clinical trials for AD and dementia patients: in phase I (ClinicalTrials.gov Identifier: NCT01729598) to test the effect of VPA on the expression of clusterin, which is a currently studied epigenetic biomarker of AD; in phase III (ClinicalTrials.gov Identifier: NCT00071721) to evaluate the effects of VPA in memory tasks of individuals with dementia; and in phase II (ClinicalTrials.gov Identifier: NCT00088387) using lithium alone or in combination with VPA (divalproex) in order to evaluate the potential decrease of altered tau protein in the spinal fluid of patients with Alzheimer’s disease. [145,179,180]. Diverse studies show that VPA enhanced H3 acetylation and consequently reduced α-synuclein-mediated toxicity and decreased pro-inflammatory mediators, in PD cell models and animals exposed to PD-promoting toxic agents, such as MPTP, rotenone, or lipopolysaccaride [162,163,164,165]. Furthermore, VPA, as well as NaB and TSA, were able to rescue dopaminergic neurons death induced by the toxic agents [167,181]. Combination of VPA with lithium enhances Ser 9 phosphorylation of GSK-3β in the lumbar spinal cord and brain. Despite the undergoing clinical trials and the beneficial effects of VPA in animal models, some human clinical studies revealed unsuccessful results or treatments required significantly high doses that result toxic and lead to unacceptable adverse effects [108,182,183]. In this regard, the pharmacogenetic profile of those patients would anticipate the interindividual tolerance levels of this treatment. 

Hydroxamic acids constitute another important class of HDAC inhibitors. Among them, the antifungal protein synthesis inhibitor Trichostatin (TSA) and the HDAC6-specific inhibitor Vorinostat (SAHA) are the most widely explored compounds for the treatment of neurodegenerative diseases [48,108,109,110,111,112,113,114,116,167,168,184,185,186].

Trichostatin (TSA) is class I HDAC inhibitor that enhances the expression of genes involved in memory consolidation, possibly by promoting the acetylation of the histone H4 [108,109,110]. Some studies also reflect restorage of memory function in APP/PS1-AD transgenic mice [48,112]. TSA also reduces neurotoxicity in α-synuclein overexpressing Drosophila models of PD, improving locomotor impairment, and reducing early mortality rates [114,116], as well as promoting H3 acetylation-mediated *GDNF* upregulation in astrocytes [168,185,186].

The HDAC6-selective inhibitor Vorinostat (SAHA) is one of the most developed HDAC inhibitors and was approved by FDA in 2006 for the treatment of advanced cutaneous T-cell lymphoma. SAHA treatment enhances basal postsynaptic excitatory, but not inhibitory, synaptic function and restores memory function in animal models of impaired learning tasks [113] and in the transgenic APPswe/PS1dE9-AD mouse [111,184].

Some benzamides, such as entinostat and W2, provided promising results as HDACi in animal models of AD. The selective HDAC1 inhibitor entinostat (MS-275) improved behavioral activities in the AD-APPPS1-21 mouse model by reducing amyloid plaque deposition and neuroinflammatory processes [187], while the mercaptoacetamide-based class II HDACi (W2) improved memory tasks and reduced tau phosphorylation rates and Aβ deposition in triple transgenic 3xTg-AD mice [188].

Several miscellaneous HDAC inhibitors are under testing for neurodegenerative diseases, especially for those involving dementia. Among this group, one of the most promising epidrugs is the Forum Pharmaceutical compound (FRM-0334), specifically tested for dementia, which inhibits a subset of class I and II human HDACs with a high efficiency (nanomolar IC50 values) and addresses the issue of crossing the blood–brain barrier [189]. FRM-0334, also called EPV-0334, promotes histone 2A, 3, and 4 acetylation in the brain and exerts a potential neuroprotective role by restoring the levels of the growth factor progranulin, which results in a significant improvement of cognitive performance in mice and rat models of frontotemporal dementia [171]. This compound is currently under phase II clinical trials in individuals with mutations in the progranulin gene diagnosed with mild to moderate frontotemporal dementia [145,153].

HDACs, and specially HDAC6, correlate with neuronal impairment and cell death, normally via microtubule destabilization. These high levels of HDAC6 frequently lead to neurodegeneration in hippocampi of AD patients. Along with SAHA, other compounds including Tubacin, Tubastatin A, and quinazolin-4-one derivatives are HDAC6-selective inhibitors with valuable potential benefits on enhancing neurite extension and reducing cell death. Tubacin (EC50 = 2.5 μM in A549 cells) exhibits 70-fold higher selectivity for HDAC6 as compared to other HDAC inhibitors in alveolar basal epithelial A549 adenocarcinoma cells. Tubacin inhibits HDAC6-targeted α-tubulin deacetylation and migration in cancer cells expressing HDAC6. Furthermore, this compound attenuates tau phosphorylation in vitro [181,190,191]. Similar to Tubacin, the affinity of the hydroxamic acid derivative, Tubastatin A, for HDAC6 is 50 to 2000-fold higher compared to other isozymes [192]. The quinazolin-4-one derivative *N*-hydroxy-3-(2-methyl-4-oxo-3-phenethyl-3,4-dihydro-quinazolin-7-yl)-acrylamide (3f) is the synthetic compound with the highest affinity and efficiency to inhibit HDAC6 in vitro (IC50, 29 nM). This compound promoted the expression of the growth-associated protein 43 which favored neurite outgrowth and enhanced the synaptic activities of PC12 and SH-SY5Y neuronal cells without toxic or mitogenic effects, and decreased zinc-mediated β-amyloid aggregation without affecting membrane channel (IC50 >10 μM) or cytochrome P450 activity (IC50 > 6.5 μM) in vitro. In addition this quinazolin-4-one derivative enhanced memory performances in AD animal models with β-amyloid-induced hippocampal lesions. [193].

#### 4.2.2. Class III HDAC (SIRT) Inhibitors

A number of studies demonstrate the beneficial effect of class III HDAC (SIRT) modulators for the treatment of several types of cancer; some of them are promising treatments for neurodegenerative disorders. Although SIRT inhibition often associates with activation of cell death pathways in multiple models of cancer, we will focus on those several SIRT inhibitors (SIRTi) that provide physiologically relevant benefits for neurodegenerative processes. Indeed, sirtuins, particularly SIRT2, favor the pathological progression of PD by promoting α-synuclein expression and aggregation. In this regard, SIRT2 inhibition rescued α-synuclein-mediated toxicity in several animal models of PD [115]. Some of these epidrugs, such as Nicotinamide, show pan-sirtuin inhibition properties, whereas other SIRT inhibitors target only SIRT1/2 or either one alone.

Among the pan inhibitors, Nicotinamide is the most widely tested for treatment of neurodegenerative diseases, especially Alzheimer’s and Huntington diseases (Table 1). Nicotinamide is a competitive and selective inhibitor of class III NAD^+^-dependent HDACs (SIRT inhibitor) used in gene regulation experiments [194]. This compound improves the stability of microtubules by reducing phosphorylated tau (at Thr231 level) and restored cognitive deficits in triple transgenic 3xTg-AD mice [195]. Nicotinamide is being currently tested, versus placebo, in two clinical trials as a potential treatment for mild to moderate AD. One of the trials completed the phase I (and phase II for placebo) (ClinicalTrials.gov Identifier: NTC00580931) and the other trial is recruiting patients for phase II (ClinicalTrials.gov Identifier: NTC03061474) [145,195,196].

SIRT2 inhibitors are also widely tested in animal models of neurodegeneration [117,197,198,199]. Among them, the brain-permeable inhibitor AK-7 displayed important neuroprotective properties in animal models of PD by improving motor functions, extending survival, and reducing alpha-synuclein aggregation [197,198].

The vinyl nitrile compound AGK2 significantly reduces tubulin deacetylation and formation of large α-synuclein inclusions, resulting in the rescue of dopaminergic neurons in vitro and in animal PD models [117]. AGK2 targets SIRT2 with a 10-fold higher selectivity as compared with the SIRTs 1 and 3 (IC_50_ of 3.5 μM), although SirReal2 is considered as the highest specific SIRT2 inhibitor, with an IC_50_ within the nM range [199]. Substrate competition chemical analyses demonstrated that this compound is able to bind and induce conformational changes in a previously unexploited binding pocket of SIRT2.

Other relevant SIRT inhibitors are sirtinol and selisistat [200,201,202,203,204]. Sirtinol is a SIRT1/2 inhibitor discovered in 2001 by a high-throughput cell-based screening and plays important roles in different physiological pathways, such as axonal protection following nerve injury or modulation of sirtuins in cardioprotection [200,201]. Selisistat was the first identified potent and cell permeable SIRT1-specific inhibitor [202,203].

#### 4.2.3. Class III HDAC (SIRT) Activators

Stress signaling pathways resulting in DNA damage and impaired DNA repair mechanisms are common hallmarks of neurodegeneration. The brain protective roles of sirtuins (SIRT), which include response to stress and activation of DNA repair pathways, site SIRT activating compounds as good potential candidates for treatment of neurodegeneration [204,205,206,207,208,209]. Resveratrol and derivatives are the most widely-tested SIRT activators in animal models of neurodegeneration.

Resveratrol is a neuroprotective compound extracted from red grapes, with important antioxidant and anti-inflammatory roles which result in the inhibition of Aβ aggregation and Aβ-induced apoptosis [210,211]. This compound might reduce miR-124 and miR-134 expressions, which would enhance cAMP response element-binding protein (CBP) levels and promote BDNF synthesis [212]. All these effects result in increased cell viability through the stabilization of Ca^2+^ homeostasis, reduction of Aβ_25–35_ neurotoxicity, and Rho-associated kinase 1 downregulation [212]. Resveratrol belongs to the family of drugs regulating GABA receptors. Much research has corroborated the importance of GABA receptors in the regulation of the neuronal pathways involved in memory and learning and, therefore, the GABAergic system has come to be seen as a promising therapeutic target for AD [213,214]. Currently, for resveratrol, four clinical trials are underway to test the potential of resveratrol in the prevention of cognitive impairment and cerebrovascular dysfunction in AD. Of these, two studies have already been completed: in phase II (ClinicalTrials.gov Identifier: NCT01504854) and phase III (ClinicalTrials.gov Identifier: NCT00678431). Of the remaining two trials, one has been withdrawn (ClinicalTrials.gov Identifier: NCT00743743) and the other is still recruiting participants (ClinicalTrials.gov Identifier: NCT02502253) [147,214] (Table 1).

Some Resveratrol structural derivatives, such as the stilbene Piceatannol, the chalcones Butein and Isoliquiritigenin, and the flavones Fisetin and Quercetin [2,215,216,217], are SIRT1 deacetylase activators that significantly extended the lifespan of *Saccharomyces cerevisiae* [218] and in *Drosophila melanogaster* S2 cells [216]. Furthermore, a diet containing 100 μM Fisetin extended Drosophila lifespan at a rate of 23%, as compared with Resveratrol, which increased lifespan of the flies at a rate of 29% [216].

### 4.3. Histone Acetyltransferase (HAT) Modulators

HAT-activating compounds, targeting CBP, p300, and p300/PCAF, would be an alternative strategy of promoting histone acetylation levels, although the poor solubility and membrane permeability of these compounds make them rather unsuitable for this purpose [2,19,26].

Importantly, a variety of chemical modifications of different nonspecific HAT inhibitors in attempts to identify enzyme-specific inhibitors, came up with the synthesis of the *N*-(4-chloro-3-trifluoromethyl-phenyl)-2-ethoxy-6-pentadecyl benzamide (CTPB), which is considered as one of the unique p300-specific activator with the capability of crossing the blood brain barrier after intraperitoneal injection [53,54].

Alternative strategies also consider natural products as HAT inhibitors [218]. The most popular HAT-inhibiting compounds are curcumin and derivatives. Other HAT-specific inhibitors include Lys-Coa targeting p300 and H3-Coa-20 for PCAF. Other HAT inhibitors are less specific but capable of permeating cells in culture [53].

Curcumin is a phytochemical compound extracted from the rhizome of *Curcuma longa*, L., used for dyspepsia, stress, and mood disorders [219]. Curcumin is a cell-permeable compound and specific inhibitor for p300/CBP, having no effect on PCAF, HDAC, and DNMT [53]. This compound protects neurons from oxidation by enhancing phase II detoxification enzymes and heme oxygenase 1 and restored mitochondrial function in brains of animal models treated with aluminum [220]. Some studies associate curcumin with a behavioral improvement, prevention of neuroinflammation, and inhibition of signaling pathways leading to Aβ aggregation and tau phosphorylation [221]. The combination of curcumin with other derivatives, such as demethoxycurcumin and bisedethoxycurcumin, which constitute turmeric [222], enhances curcumin’s properties as a potential AD treatment [222,223]. Three completed clinical trials, in phases I and II (ClinicalTrials.gov Identifier: NCT00164749), phase II (ClinicalTrials.gov Identifier: NCT00099710), and phase I (ClinicalTrials.gov Identifier: NCT01716637) [224,225,226,227] are underway to test the properties of combinations of curcumin with other natural compounds as potential treatment for AD and mild cognitive impairment [145] (Table 1). Two additional trials (ClinicalTrials.gov Identifier: NCT01811381) and (ClinicalTrials.gov Identifier: NCT02114372) are recruiting patients [147,228,229] (Table 1).

### 4.4. Modulators of Histone Methylation

#### Histone Methyltransferase Inhibitors

This subgroup of epidrugs includes histone methyltransferase and histone demethylase inhibitors. The first ones modulate gene expression and promote DNA repair by inducing histone acetylation. Despite of their potential activity, these compounds are not often good candidates for preclinical studies due to their high toxicity and low specificity in different cell lines. SAMe, one of the most important methyl donors in the body, along with l-methylfolate, also used as a DNA methylation activator, was the first HMT inhibitor used for treatment of cancer. Importantly, this compound is also submitted to a phase II clinical trial (ClinicalTrials.gov Identifier: NCT01320527) as an additive for a nutraceutical compound versus placebo for treatment of mild to moderate AD [145,147,148] and, also, in phases II and III for the treatment of depression in PD patients (ClinicalTrials.gov Identifier: NCT00070941) [145] (Table 1). Some studies indicate that SAMe induces *PSEN1* promoter methylation resulting in gene downregulation, which meliorate AD symptoms [142,143,230].

The most analyzed histone demethylase family is the Lysine-specific demethylase 1 (LSD1), which is a flavin-dependent monoamine oxidase (MAO) that can demethylate mono- and dimethylated lysines, specifically histone 3 and lysines 4 and 9 (H3K4 and H3K9) and shares catalytic sites with MAO-A and MAO-B [231]. Inhibition of these MAO catalytic sites is a current strategy for treatment of anxiety and depressive disorders, as well as neurodegenerative PD progression [232]. Tranylcypromine (2-PCPA) is the most widely analyzed and relatively potent LSD1 inhibitor in vivo (IC_50_ 20.7) that irreversibly blocks MAO A and MAO B with IC_50_ values of 2.3 and 0.95 μM and *K*_i_ values of 101.9 and 16 μM, respectively [233].

### 4.5. Non-Coding RNAs

Non-coding RNAs (ncRNAs) modulate the expression of genes involved in brain development and function. A number of diseases link with aberrant expression of those ncRNAs, which requires the implementation of new strategies that regulate ncRNA expression and function. Indeed several approaches include RNA interference as a novel and promising therapeutic strategy for the treatment of neurodegenerative diseases. These ncRNA-based treatment strategies include the use or modulation of miRNA analogs, miRNA precursors, and anti-miRNAs.

One of the most popular strategies to reduce the detrimental effects involves downregulation of pathogenic genes. This may be achieved posttranscriptional levels by RNA interference mediated by small interference RNAs (siRNAs), short-hairpin RNAs (shRNAs), and micro-RNAs (miRNAs). Altering the expression of ncRNAs targeting pathogenic genes associated with the disease may be an acceptable strategy. However, the extremely high number of gene targets and associated ncRNAs would make the approach rather difficult. In this regard, different studies suggest more specific targets that may be suitable as potential treatments. In this regard, overexpression of miR-124 and miR-195 reduce Aβ levels by targeting *BACE1* [234,235], or miR-323-3p, might reduce AD-related neuroinflammation [236]. Other ncRNAs, such as miR-34b/c, miR-132 [237,238,239,240,241], and miR-221 [237,242], are also potential biomarkers and therapeutic targets for PD. Inhibition of miR-34b/c leads to parkin and DJ-1 downregulation in SH-SY5Y cells [238], while downregulation of miR-132 results in α-synuclein accumulation [239,240]. In addition, serum levels of miR-29c, miR-146b, miR-214, and miR-221 were significantly downregulated in patients, resulting in miR-221 being a potential predictor and therapeutic target of disease [237,242]. A recent study supports the consideration of miR-221 as a potential treatment for PD due to its protective role by regulating PC12 cell viability and apoptosis by targeting phosphatase and tensin homolog (PTEN) [243].

Other strategies deal with the regulation of other ncRNAs involved in cell growth, development, and homeostasis, such as miR-485 and miR-26a [244,245]. The synaptic vesicle glycoprotein SV2A, along with miR-485, regulates neuron homeostasis by controlling the number of dendritic spines and the establishment of synapses, as well as miR-26a. Interestingly, high miR-485 levels reduce spontaneous synaptic responses, which might have implications in AD progression [245], whereas miR-26a overexpression enhances synaptic plasticity and regulates neuronal morphogenesis [244]. Indeed, miR-26a inhibition via PTEN attenuates neuronal outgrowth. Thus, PTEN suppression by miR-26a may enhance synaptic plasticity and regulate neuronal morphogenesis [244]. Another important ncRNA involved in neurodevelopment and neurodegeneration, miR-132, is considered a potential biomarker for diagnosis and treatment of PD [237,241].

Other miRNA-based approaches target the components of the epigenetic machinery and exert direct control in DNA methylation and chromatin remodeling processes [246]. These miRNAs may target DNMT inhibitors, alternatively or synergistically, such as miRNAs targeting DNMT3A (miR-29, miR-29c, miR370, and miR-450A) and DNMT3B (miR-29, miR-148, and miR-29b) induced hypomethylation-mediated enhanced expression of tumor suppression genes, which may also be achieved by miRNAs targeting EZH2 (miR-26a, miR-101, miR138, and miR-124) and decreasing histone methylation. Other miRNAs target HDACs, such as miR-449 and miR-874 for HDAC1, and miR-1 and miR-155 target HDAC4 reducing transcriptional activity of B-cell lymphoma 6. Other miRNAs, such as miR-155 and miRNA-627, reduced histone dimethylation and hypoxic gene expression [246].

### 4.6. Other Potential Epigenetic Treatments

Other promising epigenetic-based therapeutic approaches, currently submitted to preclinical studies [2,145,152,153,247,248], include the following, (i) small molecule inhibitors to chromatin-associated proteins, especially those targeting histone methyltransferases and histone demethylases; (ii) bromodomain/chromodomain inhibitors, which regulate chromatin structure and inhibit targeting gene transcription, respectively; and (iii) dietary regimens based on B vitamins and folate, in order to restore global methylation by increasing the SAMe levels in the organism, or low caloric-based regimes that might promote SIRT-mediated DNA repair mechanisms.

## 5. Neurodegeneration-Mediated DNA Methylation Patterns of Genes Involved in Drug Metabolism and Transport

Drug pharmacodynamics and pharmacokinetics influence drug response in terms of efficiency, required dosage, and toxicity. The variability of genetic and epigenetic profiles, as well as disease determinants, explain the individual differences in drug response. Pharmacogenomics accounts for 30 to 90% of the variability in pharmacokinetics and pharmacodynamics. This variability depends on polymorphic variants of five different categories of genes: (i) pathogenic genes associated with disease development or potential risk. Not all individuals carrying the same disease present the same affected pathogenic genes; (ii) genes associated with the mechanism of action of drugs (enzymes, receptors, messengers, etc.); (iii) genes associated with drug metabolism. This category includes genes associated with Phase I enzymes, such as, alcohol dehydrogenases (*ADHs*), monoamine oxidases (*MAOs*), cytochrome p450 family genes (*CYPs*), and Phase II enzymes, which include UDP glucuronosyltransferases (*UGTs*), gluthatione *S*-transferase family genes (*GSTs*), *N*-acetyltransferase (*NATs*), and sulfonotransferases (*SULTs*); (iv) genes encoding drug transporters (Phase III), such as ATP-binding cassette family members (*ABCs*), solute carrier superfamily (*SLCs*), and the solute carrier organic transporter family (*SLCOs*); and (v) pleiotropic genes involved in multiple pathways and metabolic reactions [2,3,13,15,16,19,26]. The efficiency of drug metabolizing products is influenced by genetic and epigenetic modifications on these genes [3,13,105,106].

Pharmacoepigenomics deals with the influence of epigenetic modifications on the pharmacogenomic network responsible for the pharmacokinetics and pharmacodynamics of drugs, as well as with the effects that drugs may have on the epigenetic machinery. Despite of the scarce available information on the pharmacoepigenomics of most drugs, growing evidence indicates that epigenetic changes are determinant in the pathogenesis of many medical conditions and in drug response and drug resistance [2,249,250]. The drug response varies according to polymorphic variants of genes involved in the pharmacogenomic response, as well as by epigenetic modifications in these genes that alter their expression patterns. The acquisition of drug resistance involves post-transcriptional regulators, such as RNA-binding proteins (RBPs) and miRNAs, which alter the stability and expression of genes and gene clusters involved in cell survival, proliferation, and drug metabolism [2,249,250].

The implication of cytochrome P450 enzymes (CYPs) in PD pathophysiology is fairly well-demonstrated [251], although there is no clear-cut evidence whether polymorphisms in these genes confer susceptibility to PD or what could be the effects of these polymorphisms on enzyme activity [252,253]. In the CNS, CYP2E1 colocalizes to tyrosine hydroxylase-positive neurons in the substantia nigra [254,255]. Enhanced CYP2E1 activity promoted ROS production, inhibited dopamine release in animal models, and facilitated the production of isoquinolines, structurally related to the PD-inducing toxicant MPTP, which may thus contribute to dopaminergic neurodegeneration in PD [251,252,253,256]. A genome-wide methylation analysis of PD with quantitative DNA methylation levels of 27,500 CpG sites corresponding to 14,495 genes showed a significant methylation decrease of the *CYP2E1* gene with the corresponding mRNA overexpression in brains from PD patients, suggesting that epigenetic variants of this cytochrome contribute to PD susceptibility [257] (Table 2). These results suggest that altered methylation of *CYP2E1* in PD may contribute to the individual susceptibility and help to explain the conflicting findings with regard to environmental toxins and genetic vulnerability [257].

Genome-wide DNA methylation analyses in brain and blood samples from PD patients displayed a number of epigenetic biomarkers associated with the pathological mechanisms of the disease. Importantly, authors identified concordant methylation alterations in brain and blood, suggesting that the blood might hold promise as a surrogate for brain tissue to detect DNA methylation in PD and as a source for biomarker discovery [10]. Among these biomarkers, they found altered methylation and gene expression levels in the phase II metabolizing genes encoding glutathione S-transferase *GSTT1* [10,258,259,260], *GSTTP1* [10,258,259,260], and *GSTTP2* [10,258,261], involved in conjugation of electrophiles and protection against reactive oxygen species (Table 2). Polymorphisms in these genes have been previously associated with increased risk to PD after exposure to the herbicide Paraquat [10]. Other studies found correlation in brain and blood biomarkers associated with PD progression, reinforcing the idea that detection of differential methylation events pertinent to PD pathology is feasible from blood samples [10,261,262,263,264]. Among these biomarkers, they found significant hypomethylation levels at the promoter region of the drug ABC and solute carrier organic transporter genes *ABCA3* [10,261,262], *SLC12A5* [10,263,265], and *SLC25A24* [10,264,265] (Table 2).

Several transporter genes are involved in the control of cholesterol homeostasis and influence the pathogenesis of neurodegenerative diseases. *ABCA1*, *ABCB1*, and *ABCG2* influence AD and Aβ deposition in extracellular senile plaques [266,267,268,269,270,271] (Table 2). Brain ABCA1 mediates cholesterol and phospholipid efflux and lipidates APOE to allow its interaction with Aβ and inhibits formation of Aβ deposits [272]. ABCA2, the most abundant ABC transporter in human and rodents, may regulate esterification of plasma membrane-derived cholesterol by modulation of the sphingolipid metabolism. Some authors suggest that dysregulation of *ABCA2* gene may be involved in AD pathogenesis [273]. The epigenetic machinery controls the expression of these *ABC* transporter genes through interaction with miRNAs, such as miR-33a/b-5p, miR-106b, and miR-758–5p, regulating *ABCA1* gene expression [274].

Brain DNA methylation and mRNA expression patterns in the genes coding for the ABC transporter ABCA7 and the solute carrier organic transporter SLC24A4 also associated with AD progression in a study with 740 autopsied participants older than 66 years old [262] (Table 2). The authors suggest that altered methylation in these loci might involve both Aβ and tau tangle pathologies [264]. Previous studies associate ABCA7 with the regulation of APP processing in vitro, inhibition of Aβ secretion in cultured cells, and regulation of Aβ homeostasis in the brain and deletion of *ABCA7* doubles cerebral Aβ accumulation in transgenic mouse models [275,276]. The functional relationship between SLC24A4 and AD is rather indirect. Some authors suggest that *SLC24A4* may be involved in neural development [277] or may interact with genes directly involved in AD progression, such as *BIN1* [278]. A recent study also concluded that the *ABCA7* mRNA expression level in peripheral blood may be an early diagnostic and disease progression AD biomarker regardless of the genetic polymorphism and the promoter methylation level [265].

## 6. Conclusions and Future Directions

Neurodegenerative diseases, as well as other complex multifactorial disorders, result from a complex interplay of genetic and epigenetic factors, influenced by environmental factors, which makes understanding the molecular mechanisms underlying their pathological progression difficult. Neurodegeneration involves premature events affecting cell metabolism, growth, and development only detected after several years of disease progression, when the rate of cell loss is high enough to hinder treatment possibilities. Therefore, the attempts to achieve effective treatments for those diseases have been rather unsuccessful, expensive, and limited to a symptomatic relief. It is therefore important to find diagnostic strategies for detection of neurodegenerative diseases during early, preferably asymptomatic stages, when a pharmacological intervention is still possible.

The analysis of epigenetic alterations during disease progression allows the detection of early diagnostic biomarkers of the disease. Furthermore, assuming that these epigenetic modifications are reversible and potentially targeted by pharmacological interventions, a number of epigenetic-based drugs (epidrugs), are opening a novel, promising approach for the treatment of such complex diseases [2,13,18,19,20]. However, in spite of the promising results of these epidrugs in vitro, in cell, and in animal models of neurodegeneration, only a few of them are currently submitted to clinical trials for the treatment of these diseases and none of them are yet approved by any of the main regulatory agencies [145]. A number of these epidrugs, like some DNMT inhibitors or pan HDAC inhibitors, induce toxicity and cell death due to global hypomethylation or histone acetylation, respectively, the high doses required, or the incapability to cross the blood–brain barrier [2,248,249]. Furthermore, the wide range of epigenetic alterations directly and indirectly linked to these diseases hinders the accurate selection of specific targets for effective treatments. Specific gene targeting by using ncRNAs may be a potential strategy to overcome these issues, especially by their relatively easy detection as cell-free biomarkers using noninvasive techniques that allow the tracking of the patient’s disease progression. However, most ncRNAs are not gene-specific but recognize several targets. In addition, the major problem with ncRNA approaches includes delivery systems and off-target effects. Exosomes and conjugation with cholesterol molecules may help delivery of ncRNA across the blood–brain barrier, although the detrimental effects of the moiety of these molecules to the human brain still needs to be tested [2,279,280,281,282].

Alternative strategies in order to minimize clinical complications include the development of novel natural bioproducts with neuroprotective properties and additional benefits including antioxidant, anti-inflammatory, and neurotrophic effects. E-PodoFavalin-15999 (Atremorine) is an example of a novel bioproduct obtained by means of nondenaturing biotechnological procedures from structural components of *Vicia faba* L., for the prevention and treatment of PD [17,283,284,285,286,287]. The high content of natural L-DOPA (average concentration 20 mg/g) in the composition of Atremorine provides its high dopaminergic effect on dopaminergic neurons [17,283,286,287], whereas the neuroprotective effect of this compound relies on other intrinsic constituents, such as selective neurotrophic factors [17,283]. The combination of Atremorine with conventional antiparkinsonian drugs minimizes the “wearing off” effect, as it extends the beneficial effects of the last ones with a dose reduction of 25 to 50%, which minimizes the adverse effects of the conventional antiparkinsonian compounds [17,283,286,287].

Since therapeutic outcomes are highly dependent upon the individual genomic and epigenomic profiles, personalized treatments should rely on pharmacogenetic and pharmacoepigenetic procedures to optimize therapeutics. Versatility of the epigenetic machinery allows the manipulation of epigenetic aberrations leading to drug resistance. Thus, routine procedures should incorporate pharmacoepigenetic studies for the proper evaluation of efficacy and safety issues in drug development and clinical trials. However, there is not information about long-term effects of epigenetic-based using targets without any particular cell specificity. Thus, despite substantial progress, the field of pharmacoepigenetics and the role of epigenetic modifications in human health and the treatment of disease require further investigation.

## Figures and Tables

**Table 1:** Pharmacogenetic profiles of epigenetic-based compounds currently submitted to clinical trials for the treatment of Alzheimer’s and Parkinson’s diseases.

Drug	Compound	Pharmacogenetics	Mechanisms of action	ClinicalTrials.gov ID
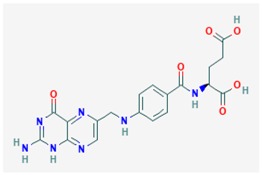	**Name:** Vitamin B9; Folic acid; Folate; 59-30-3; Folacin; Pteroylglutamic acid **IUPAC name:** (2S)-2-[[4-[(2-amino-4-oxo-1H-pteridin-6-yl)methylamino]benzoyl]amino]pentanedioic acid **Molecular formula:** C_19_H_19_N_7_O_6_ **Molecular Weight:** 441.40 g/mol **Category:** SAMe methyl donors **Targets:** SAMe	**Pathogenic genes:** *ADORA2A*, *AOX1*, *APOB*, *CDKN2A*, *COMPT* **Mechanistic genes:** *ALDH1A1*, *GSTA1*, *GSTP1*, *IL2*, *IL6*, *NAT2*, *SOD3*, *TNF*, *VGFA* **Metabolic genes:** **Substrate:***ABCG2*, *MTHFR* **Inhibitor:***ABCB1*, *ERCC2*, *MTHFR* **Inducer:***CYP2C9* **Transporter genes:** *ABCC1*, *ABCC2*, *ABCC3*, *SLC19A1*, *SLC22A8*, *SLC28A2*, *SLCOB1* **Pleiotropic genes:** *PPAR*, *TNF*, *TP53*, *VCAM1*	➢ Ameliorates memory and learning tasks in dementia patients ➢ Restores DNA methylation by increasing the SAM/SAH ratio ➢ Reduces toxicity mediated by Aβ aggregation ➢ Reduces neuroinflammation	NCT00056225-Phase III NCT01320527-Phase II NCT02457507-Phase IV
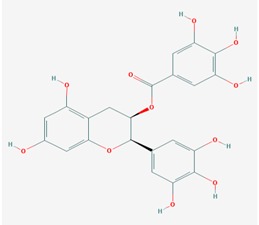	**Name:** EGCG, (−)-epigallocatechin gallate, epigallocatechin 3-gallate, tea catechin, teavigo, catechin deriv., 989-51-5 **IUPAC name:** [(2R,3R)-5,7-dihydroxy-2-(3,4,5-trihydroxyphenyl)-3,4-dihydro-2H-chromen-3-yl] 3,4,5-trihydroxybenzoate **Molecular formula:** C_22_H_18_O_11_ **Molecular Weight:** 458.37 g/mol **Category:** DNMT inhibitors **Targets:** DNMT1	**Pathogenic genes:** *APP*, *BACE1*, *CDX2*, *EGFR*, *FAS PIK3CA*, *ROS1* **Mechanistic genes:** *APP*, *BACE1*, *BMP2*, *CDX2*, *CHRNA7*, *ECEs*, *EGFR*, *IRS1*, *PIK3CA*, *ROS1* **Metabolic genes:** **Inhibitor:***SOD* **Transporter genes:** *CD36*, *SLC5A1*, *SLC27A4*, *SLCO1B1*, *SLCO1B3* **Pleiotropic genes:** *ACACA*, *CHRNA7*, *SCD*	➢ Prevents misfolded proteins from fibrillization ➢ Restores respiratory rates and membrane potential in isolated mitochondria from hippocampus, cortex, and striatum ➢ Activates α7 nicotinic acetylcholine receptor (α7 nAChR) signaling cascade ➢ Restores *Bcl2* expression, preventing cell death in Aβ-treated neurons	NTC00951834-Phases II, III
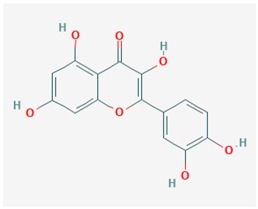	**Name:** Quercetin; Sophoretin; Quercetol; Meletin; Xanthaurine; Quercitin; 3,3′,4′,5,7-Pentahydroxyflavone **IUPAC name:** 2-(3,4-dihydroxyphenyl)-3,5,7-trihydroxychromen-4-one **Molecular formula:** C_15_H_10_O_7_ **Molecular Weight:** 302.24 g/mol **Category:** DNMT inhibitors **Targets:** DNMT1	**Pathogenic genes:** *IL1R*, *NFkB*, *Ccl8*, *IKK*, *STAT3*, *CD4*, *CDK2*, *IL2* **Mechanistic genes:** *MTND4*, *CDKN2A*, *PRDX4*, *DIO2*, *HSD17B1*, *MSH2*, *GSS*, *COMT*, *FOS*, *CRP*, *NR1I3*, *PON1* **Metabolic genes:** **Substrate:***UGT1A1*, *UGT1A3*, *GSTT1*, *CYP2J2*, *GSTK1*, *CYP2C8*, *CYP1A1*, *CYP1A2*, *CYP1B1*, *GSTA1*, *CYP19A1* **Inhibitor:***SULT1E1* **Transporter genes:** *ABCB1*, *ABCG2*	➢ Modulates immune system ➢ Exerts a positive effect in support of cognitive function	NCT01716637-Phase I
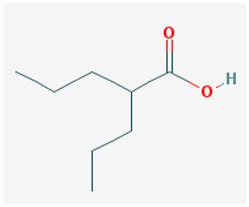	**Name:** Valproic acid, 2-propylpentanoic acid, depakene, depakine, ergenyl, dipropylacetic acid, mylproin, convulex, myproic acid **IUPAC name:** 2-propylpentanoic acid **Molecular formula:** C_8_H_16_O_2_ **Molecular Weight:** 144.21 g/mol **Category:** HDAC inhibitors **Targets:** Class I HDAC; Class II HDAC	**Pathogenic genes:** *CREB1*, *IL6*, *LEP*, *SCN2A*, *TGFB1*, *TNF*, *TRNK* **Mechanistic genes:** *ABAT*, *CDK5*, *GSK3B*, *HDAC1*, *HDAC2*, *HDAC3*, *HDAC8*, *HDAC9*, *LEP*, *LEPR*, *SCNs*, *SMN2* **Metabolic genes:** **Substrate:***CYP2A6* (major), *CYP2C9* (major), *CYP4B1* (major), *CYP1A1* (minor), *CYP2B6* (minor), *CYP2C19* (minor), *CYP2E1* (minor), *CYP3A4* (minor), *CYP4F2* (minor), *ABCB1* (minor), *UGT1A4*, *UGT1A6*, *UGT1A8*, *UGT1A10*, *UGT2B7* **Inhibitor:** *ABCB1*, *ACADSB*, *AKR1A1*, *CYP2C9* (strong), *CYP2A6* (moderate), *CYP2C19* (moderate), *CYP3A4* (moderate), *CYP2D6* (weak), *HDAC1*, *HDAC2*, *HDAC3*, *HDAC8*, *HDAC9*, *UGT1A9*, *UGT2B1*, *UGT2B7* **Inducer:** *ABCB1*, *AKR1C4*, *CASR*, *CYP2A6*, *CYP2B6*, *CYP3A4*, *CYP7A1*, *MAOA*, *NR1I2*, *SLC5A5*, *SLC6A2*, *SLC12A3*, *SLC22A16* **Transporter genes:** *ABCB1*, *ABCC2*, *ABCG1*, *ABCG2*, *SCNs*, *SLC5A5*, *SLC6A2*, *SLC12A3*, *SLC22A16* **Pleiotropic genes:** *ABL2*, *AGPAT2*, *ASL*, *ASS1*, *CDK4*, *CHRNA1*, *COL1A1*, *CPS1*, *CPT1A*, *DRD4*, *FMR1*, *FOS*, *HBB*, *HFE*, *HLA-A*, *HLA-B*, *ICAM1*, *IFNG*, *IL6*, *IL10*, *LEPR*, *NAGS*, *NR3C1*, *OTC*, *PTGES*, *STAT3*, *TGFB1*, *TNF*, *TP53*	➢ Promotes the expression of brain-derived neurotrophic factors involving neuronal growth, survival, and synaptic plasticity ➢ Induces the expression of the heat-shock protein Hsp70 ➢ Reduces Aβ production and aggregation in AD cells and animal models, through inhibition of γ-secretase cleavage of *APP* ➢ Improves memory tasks by increasing histone H4 acetylation, in combination with NaB and SAHA ➢ Reduces α-synuclein-mediated toxicity (via H3 acetylation) and decreased pro-inflammatory mediators, in PD cell models and animals exposed to toxicant agents ➢ Rescues dopaminergic neurons death induced by the toxic agents ➢ Combination of VPA with lithium enhances Ser 9 phosphorylation of GSK-3β in the lumbar spinal cord and brain.	NTC01729598-Phase I NTC00088387-Phase II NTC00071721-Phase III
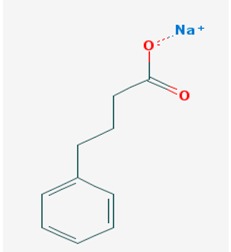	**Name:** sodium phenylbutyrate, buphenyl, 4-phenylbutiric acid, 4-phenylbutonoic acid, benzenebutanoic acid, benzenebutyric acid, butyric acid **IUPAC name:** sodium;4-phenylbutanoate **Molecular formula:** C_10_H_11_NaO_2_ **Molecular Weight:** 186.182909 g/mol **Category:** HDAC inhibitors **Targets:** Class I HDAC, Class IIa HDAC, Class IIb HDAC	**Pathogenic genes:** *ARG1*, *ASS1*, *BCL2*, *CPS1*, *NAGS*, *OTC* **Mechanistic genes:** *BCL2*, *BDNF*, *EDN1*, *HDACs*, *HSPA8*, *ICAM1*, *NFKB2*, *NT3*, *VCAM1* **Metabolic genes:** **Inhibitor:***HDACs* **Inducer:***ARG1*, *CFTR*, *CYP2B6*, *NFKB2* **Transporter genes:** *CFTR* **Pleiotropic genes:** *ASL*, *BDNF*, *VCAM1*	➢ Promotes the expression of genes involved in synaptic plasticity (via histone acetylation) ➢ Restores memory and learning functions in AD transgenic mice by reducing tau phosphorylation ➢ Reduces Aβ accumulation, and restores memory function in transgenic AD mice ➢ Protects dopaminergic neurons of mice exposed to toxicant agents	NCT03533257-Phase II NCT02046434-Phase I
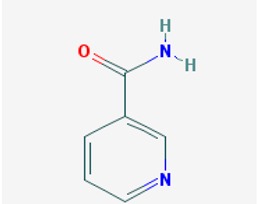	**Name:** Nicotinamide, niacinamide, vitamin PP, aminicotin, nicotinic acid amide, amixicotyn, 3-pyridinecarboxamide, papulex, nicotylamide **IUPAC name:** pyridine-3-carboxamide **Molecular formula:** C_6_H_6_N_2_O **Molecular Weight:** 122.12 g/mol **Category:** SIRT inhibitors **Targets:** class III HDAC (SIRT1-7)	**Pathogenic genes:** *IL6*, *IL8*, *PTGS2*, *TNF* **Mechanistic genes:** *ARTs*, *CAT*, *CLOCK*, *FOXO3*, *GPXs*, *IL6*, *IL8*, *PARP1*, *PTGS2*, *SIRT1*, *SOD1*, *TNF* **Metabolic genes:** **Inhibitor:***CYP2D6*, *CYP3A4*, *CYP2E1*, *SIRT1-7* **Pleiotropic genes:** *CAT*, *PARP1*	➢ Improves the stability of microtubules by reducing phosphorylated tau in triple transgenic 3xTg-AD mice ➢ restores cognitive deficits in triple transgenic 3xTg-AD mice	NTC00580931-Phases I, II NTC03061474-Phase II
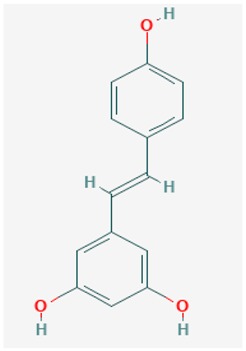	**Name:** Resveratrol, *trans*-resveratrol, 501-36-0, 3,4′,5-trihydroxystilbene, (E)-resveratrol, resvida **IUPAC name:** 5-[(E)-2-(4-hydroxyphenyl)ethenyl]benzene-1,3-diol **Molecular formula:** C_14_H_12_O_3_ **Molecular Weight:** 228.24 g/mol **Category:** SIRT inhibitors **Targets:** class III HDAC (SIRT1)	**Pathogenic genes:** *BCL2*, *CAV1*, *ESR1*, *ESR2*, *GRIN2B*, *NOS3*, *PTGS2*, *TNFRSF10A*, *TNFRSF10B* **Mechanistic genes:** *APP*, *ATF3*, *BAX*, *BAK1*, *BBC3*, *BCL2*, *BCL2L1*, *BCL2L11*, *BIRC5*, *CASP3*, *CAV1*, *CFTR*, *ESR1*, *ESR2*, *GRIN1*, *GRIN2B*, *HTR3A*, *NFKB1*, *NOS3*, *PMAIP1*, *PTGS1*, *PTGS2*, *SIRT1*, *SIRT3*, *SIRT5*, *SRC*, *TNFRSF10A*, *TNFRSF10B*, *TRPs* **Metabolic genes:** **Substrate:***CYP1A1*, *CYP1A2*, *CYP1B1*, *CYP2E1*, *GSTP1*, *PTGS1*, *PTGS2* **Inhibitor:***CYP1A1*, *CYP1B1*, *CYP2C9*, *CYP2D6*, *CYP3A4*, *NQO2* **Inducer:***CYP1A2*, *SIRT1* **Transporter genes:** *ABCC1*, *ABCC2*, *ABCC3*, *ABCC4*, *ABCC8*, *ABCG1*, *ABCG2*, *CFTR*, *TRPs*	➢ Neuroprotective role through inhibition of Aβ aggregation, along with anti-oxidative and anti-inflammatory pathways ➢ Improves long-term memory formation by promoting SIRT1 activity and inhibiting Aβ-induced apoptosis ➢ Upregulates cAMP response element-binding protein (CBP) levels and promotes synthesis of neurotrophic factors by downregulation of miR-124 and miR-134 expression	NCT01504854-Phase II NCT00678431-Phase III NCT00743743-withdrown NCT02502253-Phase I
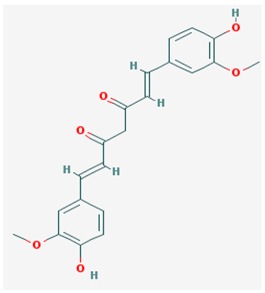	**Name:** Curcumin, diferuloylmethane, turmeric yellow, turmeric, gelbwurz, kacha haldi, curcuma, haldar, souchet **IUPAC name:** (1E,6E)-1,7-bis(4-hydroxy-3-methoxyphenyl)hepta-1,6-diene-3,5-dione **Molecular formula:** C_21_H_20_O_6_ **Molecular Weight:** 368.38 g/mol **Category:** HAT inhibitors **Targets:** HATs	**Pathogenic genes:** *BACE1*, *CCND1*, *CDH1*, *GSK3B*, *IL1A*, *IL6*, *JUN*, *MSR1*, *PSEN1*, *PTGS2*, *SNCA*, *SREBF1*, *TNF* **Mechanistic genes:** *AKT1*, *PRKAs*, *BACE1*, *CCND1*, *CDH1*, *CDKs*, *CRM1*, *CTNNB1*, *EGF*, *GSK3B*, *HDACs*, *HIF1A*, *IL1A*, *IL6*, *JUN*, *MMPs*, *MSR1*, *NFKB1*, *NOS2*, *PDGFRs*, *PSEN1*, *PTGS2*, *SNCA*, *SOCS1*, *SOCS3*, *SREBF1*, *STAT3*, *TNF*, *VEGFA* **Metabolic genes:** **Inhibitor:***CYP2C8*, *CYP2C9*, *EP300* **Inducer:***CYP2C8*, *CYP2C9*, *CYP2D6*, *CYP3A4* **Transporter genes:** *ABCA1*, *SNCA* **Pleiotropic genes:** *CTNNB1*, *MSR1*	➢ Prevents oxidation by promoting heme oxygenase 1 and Phase II detoxification enzymes in neurons ➢ Enhances mitochondrial metabolism in brains of rats treated with aluminum ➢ Prevents neuroinflammation, Aβ-mediated cell signaling disturbances, and tau phosphorylation ➢ Combination of curcumin with other derivatives, constitute the turmeric, which improves the behavioral symptoms of AD	NTC00164749-Phases I, II NTC00099710-Phase II NTC01716637-Phase I NTC01811381-Recruting NTC02114372-Recruting
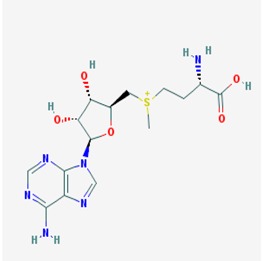	**Name:** S-adenosylmethionine, ademetionine, AdoMet, donamet, methioninyladenylate, S-adenosyl-l-methionine, SAM-e **IUPAC name:** [(3S)-3-amino-3-carboxypropyl]-[[(2S,3S,4R,5R)-5-(6-aminopurin-9-yl)-3,4-dihydroxyoxolan-2-yl]methyl]-methylsulfanium **Molecular formula:** C_15_H_23_N_6_O_5_S^+^ **Molecular Weight:** 399.45 g/mol **Category:** HMT inhibitors **Targets:** HMTs	**Pathogenic genes:** *AKT*, *ERK*, *GNMT*, *MAT1A*, *PSEN1* **Mechanistic genes:** *AMD1*, *CAT*, *CBS*, *GCLC*, *GNMT*, *GSS*, *NOS2*, *ROS1*, *STAT1*, *TNF* **Metabolic genes:** **Substrate:***COMT*, *GNMT*, *TPMT*, *SRM* **Inhibitor:***ABCB1*, *CYP2E1*, *NOS2* **Transporter genes:** *SLC25A26* **Pleiotropic genes:** *CAT*, *TNF*	➢ One of the main methyl donors in the body, as well as DNA and histone methylation activator ➢ Restores global DNA and gene specific methylation levels resulting in neuroprotection, improved memory functions, and reduced AD and PD symptoms	NTC01320527-Phase II NTC00070941-Phases II, III

ABAT: 4-aminobutyrate aminotransferase; ABCA1: ATP-binding cassette, subfamily A, member 1; ABCB1: ATP-binding cassette, subfamily B, member 1; ABCC1: ATP-binding cassette, subfamily C, member 1; ABCC2: ATP-binding cassette, subfamily C, member 2; ABCC3: ATP-binding cassette, subfamily C, member 3; ABCC4: ATP-binding cassette, subfamily C, member 4; ABCC8: ATP-binding cassette, subfamily C, member 8; ABCG1: ATP-binding cassette, subfamily G, member 1; ABCG2: ATP-binding cassette, subfamily G, member 2; ABL2: c-abl proto-oncogene 2, non-receptor tyrosine kinase; ACACA: acetyl-CoA carboxylase alpha; ACADSB: acyl-CoA dehydrogenase, short/branched chain; ADORA2A: adenosine 2A2 receptor; AGPAT2: 1-acylglycerol-3-phosphate *O*-acyltransferase 2; AKR1A1: aldo-keto reductase family 1 member A1; AKT1: AKT serine/threonine kinase 1; ALDH1A1: Aldehyde dehydrogenase 1 family, member A1; AMD1: adenosylmethionine decarboxylase 1; AOX1: aldehyde oxidase 1; APOB: apolipoprotein B; APP: amyloid beta precursor protein; ARG1: arginase 1; ARTS: ADP ribosyltransferases; ASL: argininosuccinate lyase; ASS1: argininosuccinate synthase 1; ATF3: activating transcription factor 3; BACE1: beta-secretase 1; BAK1: BCL2 antagonist/killer 1; BAX: BCL2 associated X, apoptosis regulator; BBC3: BCL2 binding component 3; BCL2: B-cell lymphoma 2, apoptosis regulator; BCL2L1: BCL2 like 1; BCL2L11: BCL2 like 11; BDNF: brain-derived neurotrophic factor; BIRC5: baculoviral IAP repeat containing 5; BMP2: bone morphogenetic protein 2; CASP3: caspase 3; CASR: calcium sensing receptor; CAT: catalase; CAV1: caveolin 1; CBS: cystathionine-beta-synthase; Ccl8: C-C motif chemokine ligand 8; CCND1: cyclin D1; CD36: CD36 molecule; CD4: CD4 molecule; CDH1: cadherin 1; CDK: cyclin-dependent kinase; CDK2: cyclin-dependent kinase 2; CDK4: cyclin-dependent kinase 4; CDK5: cyclin-dependent kinase 5; CDKN2A: cyclin-dependent kinase inhibitor 2A; CDX2: caudal type homeobox 2; CFTR: cystic fibrosis transmembrane conductance regulator; CHRNA1: cholinergic receptor nicotinic alpha 1 subunit; CHRNA7: cholinergic receptor nicotinic alpha 7 subunit; CLOCK: circadian locomotor output cycles kaput; COL1A1: collagen type I alpha 1 chain; COMT: catechol-*O*-methyltransferase; CPS1: carbamoyl-phosphate synthase 1; CPT1A: carnitine palmitoyltransferase 1A; CREB1: cAMP responsive element binding protein 1; CRM1: exportin CRM1; CRP: C-reactive protein; CTNNB1: catenin beta 1; CYP19A1: cytochrome P450 family 19 subfamily A member 1; CYP1A1: cytochrome P450 family 1 subfamily A member 1; CYP1A2: cytochrome P450 family 1 subfamily A member 2; CYP1B1: cytochrome P450 family 1 subfamily B member 1; CYP2A6: cytochrome P450 family 2 subfamily A member 6; CYP2B6: cytochrome P450 family 2 subfamily B member 6; CYP2C19: cytochrome P450 family 2 subfamily C member 19; CYP2C8: cytochrome P450 family 2 subfamily C member 8; CYP2C9: cytochrome P450 family 2 subfamily C member 9; CYP2D6: cytochrome P450 family 2 subfamily D member 6; CYP2E1: cytochrome P450 family 2 subfamily E member 1; CYP2J2: cytochrome P450 family 2 subfamily J member 2; CYP3A4: cytochrome P450 family 3 subfamily A member 4; CYP4B1: cytochrome P450 family 4 subfamily B member 1; CYP4F2: cytochrome P450 family 4 subfamily F member 2; CYP7A1: cytochrome P450 family 7 subfamily A member 1; DIO2: iodothyronine deiodinase 2; DR4: drought-repressed 4; ECEs: endothelin converting enzymes; EDN1: endothelin 1; EGF: epidermal growth factor; EGFR: epidermal growth factor receptor; EP300: E1A binding protein p300; ERCC2: excision repair, complementing defective, in Chinese hamster, 2; ERK: extracellular regulated MAP kinase; ESR1: estrogen receptor 1; ESR2: estrogen receptor 1; FAS: Fas (TNF receptor superfamily member 6); FMR1: fragile X mental retardation 1; FOS: FBJ osteosarcoma oncogene; FOXO3: forkhead box O3; GCLC: glutamate-cysteine ligase catalytic subunit; GNMT: glycine *N*-methyltransferase; GPX: phage tail protein; GRIN1: glutamate ionotropic receptor NMDA type subunit 1; GRIN2B: glutamate ionotropic receptor NMDA type subunit 2B; GSK3B: glycogen synthase kinase 3 beta; GSS: glutathione synthetase; GSTA1: glutathione S-transferase alpha 1; GSTK1: glutathione S-transferase kappa 1; GSTP1: glutathione S-transferase pi 1; GSTT1: glutathione S-transferase theta 1; HATs: Histone acetyltransferases; HBB: hemoglobin subunit beta; HDACs: histone deacetylases; HDAC 1-9: histone deacetylases 1–9; HFE: hemochromatosis; HIF1A: hypoxia inducible factor 1 alpha subunit; HLA-A: major histocompatibility complex, class I, A; HLA-B: major histocompatibility complex, class I, B; HMTs: Histone Methyl Transferases; HSD17B1: hydroxysteroid 17-beta dehydrogenase 1; HSPA8: heat-shock 70-KD protein 8; HTR3A: 5-hydroxytryptamine receptor 3A; ICAM1: intercellular adhesion molecule 1; IFNG: interferon gamma; IKK: I-kappaB kinase beta; IL10: interleukin 10; IL1A: interleukin 1A; IL1R: interleukin receptor; IL2: interleukin 2; IL6: interleukin 6; IL8: interleukin 8; IRS1: insulin receptor substrate 1; JUN: Jun proto-oncogene, AP-1 transcription factor subunit; LEP: leptin; LEPR: leptin receptor; MAOA: monoamine oxidase A; MAT1A: methionine adenosyltransferase 1A; MMP: matrix metalloproteinase; MSH2: mutS homolog 2; MSR1: macrophage scavenger receptor 1; MTHF: 5,10 methylenetetrahydrofolate; MTHFR: 5,10 methylenetetrahydrofolate receptor; MTND4: mitochondrially encoded NADH dehydrogenase 4; NAGS: *N*-acetylglutamate synthase; NAT2: *N*-actyltransferase 2; NFkB: nuclear factor kappa-B; NFkB1: nuclear factor kappa B subunit 1; NFkB2: nuclear factor kappa B subunit 2; NOS2: nitric oxide synthase 2; NOS3: nitric oxide synthase 3; NQO2: *N*-ribosyldihydronicotinamide:quinone reductase 2; NR1I2: nuclear receptor subfamily 1 group I member 2; NR1I3: nuclear receptor subfamily 1 group I member 3; NR3C1: nuclear receptor subfamily 3 group C member 1; NT3: neurotrophin 3; OTC: ornithine carbamoyltransferase; PARP1: poly(ADP-ribose) polymerase 1; PDGFR: platelet derived growth factor receptor; PIK3CA: phosphatidylinositol-4,5-bisphosphate 3-kinase catalytic subunit alpha; PMAIP1: phorbol-12-myristate-13-acetate-induced protein 1; PON1: paraoxonase 1; PPAR: peroxisome proliferator-activated receptor; PRDX4: peroxiredoxin 4; PRKA: serine protein kinase PrkA; PSEN1: presenilin 1; PTGS1: prostaglandin-endoperoxide synthase 1; PTGS2: prostaglandin-endoperoxide synthase 2; ROS1: ROS proto-oncogene 1, receptor tyrosine kinase; SAMe: S-adenosylmethionine; SCD: stearoyl-CoA desaturase; SCN2A: sodium voltage-gated channel alpha subunit 2; SIRT1-7: sirtuins 1–7; SLC12A3: solute carrier family 12 member 3; SLC22A16: solute carrier family 22 member 16; SLC25A26: solute carrier family 25 member 26; SLC27A4: solute carrier family 27 member 4; SLC5A1: solute carrier family 5 member 1; SLC5A5: solute carrier family 5 member 5; SLC6A2: solute carrier family 6 member 2; SLC19A1: solute carrier family 19 member 1; SLC22A8: solute carrier family 22 member 8; SLC28A2: solute carrier family 28 member 8; SLCO1B1: solute carrier organic anion transporter family member 1B1; SLCO1B3: solute carrier organic anion transporter family member 1B3; SMN2: survival of motor neuron 2, centromeric; SNCA: synuclein alpha; SOCS1: suppressor of cytokine signaling 1; SOCS3: suppressor of cytokine signaling 3; SOD1: superoxide dismutase 1; SOD3: superoxide dismutase 3; SRC: SRC proto-oncogene, non-receptor tyrosine kinase; SREBF1: sterol regulatory element binding transcription factor 1; SRM: spermidine synthase; STAT1: signal transducer and activator of transcription 1; STAT3: signal transducer and activator of transcription 3; SULT1E1: sulfotransferase family 1E member 1; TGFB1: transforming growth factor beta 1; THF: Tetrahydrofolate; TNF: tumor necrosis factor; TNFRSF10A: TNF receptor superfamily member 10A; TNFRSF10B: TNF receptor superfamily member 10B; TP53: tumor protein p53; TPMT: thiopurine S-methyltransferase; TRNK: mitochondrially encoded tRNA lysine; UGT1A1: UDP glucuronosyltransferase family 1 member A1; UGT1A10: UDP glucuronosyltransferase family 1 member A10; UGT1A3: UDP glucuronosyltransferase family 1 member A3; UGT1A4: UDP glucuronosyltransferase family 1 member A4; UGT1A6: UDP glucuronosyltransferase family 1 member A6; UGT1A8: UDP glucuronosyltransferase family 1 member A8; UGT1A9: UDP glucuronosyltransferase family 1 member A9; UGT2B1: UDP glucuronosyltransferase family 2 member B1; UGT2B7: UDP glucuronosyltransferase family 2 member B7; VCAM1: vascular cell adhesion molecule 1; VEGFA: vascular endothelial growth factor A.

**Table 2:** Epigenetic modifications in genes involved in the pharmacogenomic response to drugs associated with the onset and/or progression of Alzheimer’s and Parkinson’s diseases.

Category	Gene	Locus	OMIM	Pathology	Epigenetic changes
Phase I Drug Metabolizers	*CYP2E1*	10q26.3	124,040	Parkinson’s Disease	Hypomethylated Up-regulated mRNA
Phase II Drug Metabolizers	*GSTT1*	22q11.23	600,436	Parkinson’s Disease	Hypomethylated Upregulated mRNA
	*GSTTP1*	22q11.23	600,436	Parkinson’s Disease	Hypermethylated Downregulated mRNA
	*GSTTP2*	22q11.23	600,436	Parkinson’s Disease	Hypermethylated Downregulated mRNA
Phase III Drug Transporters	*ABCA1*	9q31.1	600,046	Alzheimer’s Disease	Hypermethylated Downregulated mRNA
	*ABCB1*	7q21.12	171,050	Alzheimer’s Disease	Hypermethylated Downregulated mRNA
	*ABCG2*	4q22.1	603,756	Parkinson’s Disease	Hypermethylated Downregulated mRNA
	*ABCA3*	16p13.3	601,615	Parkinson’s Disease	Hypomethylated Upregulated mRNA
	*ABCA7*	19p13.3	605,414	Alzheimer’s Disease	Hypomethylated Upregulated mRNA
	*SLC12A5*	20q13.12	606,726	Parkinson’s Disease	Hypomethylated Upregulated mRNA
	*SLC24A4*	14q32.12	609,840	Alzheimer’s Disease	Hypomethylated Upregulated mRNA
	*SLC25A24*	1p13.3	608,744	Parkinson’s Disease	Hypomethylated Upregulated mRNA

ABCA1: ATP-binding cassette, subfamily A (ABCA), member 1; ABCA3: ATP-binding cassette, subfamily A (ABCA), member 3; ABCA7: ATP binding cassette subfamily A member 7; ABCB1: ATP-binding cassette, subfamily B (ABCB), member 1; ABCG2: ATP-binding cassette, subfamily G (ABCG), member 2; CYP2E1: cytochrome P450 family 2 subfamily E member 1; GSTT1: Glutathione S-transferase theta 1; GSTTP1: Glutathione S-transferase theta pseudogene 1; GSTTP2: Glutathione S-transferase theta pseudogene 2; SLC12A5: Solute carrier family 12 (potassium/chloride transporter), member 5; SLC24A4: solute carrier family 24 member 4; SLC25A24: solute carrier family 25 member 24.
